# Detection and Characterization of Phosphorylation, Glycosylation, and Fatty Acid Bound to Fetuin A in Human Blood

**DOI:** 10.3390/jcm10030411

**Published:** 2021-01-22

**Authors:** Markéta Kovářová, Hubert Kalbacher, Andreas Peter, Hans-Ulrich Häring, Triantafyllos Didangelos, Norbert Stefan, Andreas Birkenfeld, Erwin Schleicher, Konstantinos Kantartzis

**Affiliations:** 1 Department for Diagnostic Laboratory Medicine, Institute for Clinical Chemistry and Pathobiochemistry, 72076 Tübingen, Germany; kovarova-marketa@seznam.cz (M.K.); andreas.peter@med.uni-tuebingen.de (A.P.); 2 Institute for Diabetes Research, and Metabolic Diseases of the Helmholtz Center Munich, at the Eberhard-Karls-University of Tübingen, 72076 Tübingen, Germany; hu.haering@gmail.com (H.-U.H.); norbert.stefan@med.uni-tuebingen.de (N.S.); andreas.birkenfeld@med.uni-tuebingen.de (A.B.); konstantinos.kantartzis@med.uni-tuebingen.de (K.K.); 3 German Center for Diabetes Research (DZD), 72076 Tübingen, Germany; 4 Interfaculty Institute of Biochemistry, at the Eberhard-Karls-University Tübingen, 72076 Tübingen, Germany; hubert.kalbacher@uni-tuebingen.de; 5 Department of Internal Medicine IV, Division of Endocrinology, Diabetology and Nephrology, University of Tübingen, 72076 Tübingen, Germany; 6 Diabetes Center, 1st Propaedeutic Department of Internal Medicine, Medical School, “AHEPA” Hospital, Aristotle University of Thessaloniki, 54636 Thessaloniki, Greece; didang@auth.gr

**Keywords:** fetuin A, phosphorylation, glycosylation, fatty acid bound to fetuin A, fatty liver, obesity, FAM20C

## Abstract

The hepatokine fetuin A (Fet A) has been associated with diverse pathological states such as insulin resistance, type 2 diabetes, macrovascular disease, and systemic ectopic and vascular calcification. Fet A may also play a role in tumor growth and metastasis. The biological activity of Fet A may be affected by various modifications, including phosphorylation, O- and N-glycosylation and fatty acid binding. We developed an antibody-based assay for the detection of Fet A phosphorylated at serine 312. Fatty acid pattern was determined by gas chromatography. Using the antibody, we found that the phosphorylation was stable in human plasma or serum at room temperature for 8 h. We observed that Fet A is present in several glycosylation forms in human plasma, but the extent of Ser^312^ phosphorylation was not associated with glycosylation. The phosphorylation pattern did not change during an oral glucose tolerance test (0–120 min). We further found that human Fet A binds preferentially saturated fatty acids (>90%) at the expense of mono- and poly-unsaturated fatty acids. Our results indicate that different molecular species of Fet A are present in human plasma and that these different modifications may determine the different biological effects of Fet A.

## 1. Introduction

In human adults, fetuin A (Fet A, synonymous with α2-Heremanns-Schmid-glycoprotein) is expressed and secreted into the circulation predominantly by the liver [1,2]. Although present in high concentrations in fetal plasma, Fet A levels are rather low in healthy adults ranging from 250–600 µg/mL; reported levels depend to some extent on the assay [3]. Fet A is a ~64 kDa glycoprotein, which consists of an A-chain (282 aa), a B-chain (182 aa) and a connecting peptide (40 aa) harboring the major phosphorylation site [3,4]. Using mass spectrometry Haglund et al. [5] found that circulating plasma Fet A (commercial Fet A preparation) is predominately phosphorylated at Ser^312^ (approximately 20%). Furthermore, Fet A contains two N-linked (Asp 138 and Asp 158) and two O-linked glycosylation sites (Thr 238 and Thr 252) further emphasizing the large heterogeneity of human fetuin A [6].

Fet A exerts numerous biological effects. Several reports describe that Fet A acts as systemic inhibitor of extraosseous calcification serving as transport protein for calcium and phosphate [1,7]. Deletion of Fet A results in massive ectopic tissue and vascular calcification [7]. Furthermore, low Fet A levels have been associated with decreased survival of renal patients [8]. Numerous reports implicate Fet A also in cell adhesion and tumor growth. A previous report has extensively reviewed the role of Fet A in tumor progression and metastasis [9].

Apart from Fet A’s role as an inhibitor of ectopic calcification, reports from several groups suggested that elevated Fet A may play a role in metabolic diseases, including the metabolic syndrome and insulin resistance [10,11,12,13,14]. In a cohort of well-characterized individuals at risk to develop type 2 diabetes, Stefan et al. demonstrated that circulating serum Fet A levels are correlated with whole body insulin resistance and liver fat [15] and that Fet A together with free fatty acids may predict insulin resistance in humans [16]. Studies by Mori et al. [17] and the Heart and Soul study indicate that Fet A is associated with insulin resistance, the metabolic syndrome, and an atherogenic lipid profile [18]. In the same line, Weikert et al. found an association of elevated plasma Fet A levels with an increased risk of myocardial infarction and ischemic stroke [19]. In a recent meta-analysis of 27 case-control and 5 cohort studies, a significant relationship between the circulating fetuin A levels and type 2 diabetes mellitus risk was reported [20]. Data from in vivo animal studies support the view that Fet A is involved in the regulation of metabolism. Fetuin A null mice show improved insulin sensitivity, resistance to weight gain and are protected against obesity and insulin resistance associated with aging [21].

On the molecular level, Auberger et al. [22] were the first to report that Fet A inhibits insulin signaling at the tyrosine kinase level. Accordingly, further studies showed that Fet A is interfering with insulin receptor kinase activity [23,24]. However, the exact molecular mechanism how Fet A interferes with the insulin signaling cascade remains controversial. Srinivas et al. [25] reported that recombinant human Fet A (rhFet A) inhibited insulin-stimulated mitogenic pathway via the Ras-Raf-MAPK arm without affecting metabolic signaling. In contrast, Goustin et al. showed that Fet A blocks the metabolic arm of insulin action through its interaction with the ß-subunit of the insulin receptor [26]. Two reports investigating the role of the phosphorylated form of Fet A found that only this form inhibits insulin receptor kinase activity [22,27]. A recent report further indicates that phosphorylated Fet A (pFet A), but not dephosphorylated Fet A, inhibits insulin-induced glucose uptake and glycogen synthesis. Furthermore, the data indicates that pFet A, but not total Fet A is associated with BMI, waste circumference, total body fat, serum glucose, serum insulin, and insulin resistance [6]. These partly controversial findings suggest that the molecular Fet A forms responsible for the biological effects are not well defined yet.

To clarify which of the molecular species of Fet A represents the physiologically most active form, we generated monospecific antibodies for detecting and quantifying pFet A and used them to investigate whether pFet A remains stable under various clinical laboratory conditions, as well as whether phosphorylation is affected by the degree of glycosylation. Last, since Fet A interacts with fatty acids to bind to toll-like receptor (TLR) 4 to trigger an inflammatory cellular response [28], we studied the pattern of fatty acids bound to Fet A.

## 2. Materials and Methods

Fetuin A (Fet A) = alpha 2 − HS Glycoprotein from human plasma (G0615), purity > 90% (Sigma-Aldrich, Munich, Germany), recombinant Fet A (rh Fet A) from baculovirus-insect cells from L. Schomburg (Humboldt University Berlin, Berlin, Germany), Fet A antibody (Abcam, Cambridge, UK), Fet A Enzyme-Linked Immunosorbent Assay (ELISA) (BioVendor, Brno, Czech Republic), recombinant human FAM20C protein (R&D Systems Inc., Minneapolis, MN, USA), Kinase assay buffer I (Signal Chem, Richmond, BC, Canada), protein deglycosylation Mix II (New England Biolab, Frankfurt am Main, Germany), infrared fluorescent dye secondary antibodies (anti-mouse and anti-rabbit), ChameleonTM Duo Pre-stained Protein Ladder (LI-COR, Lincoln, NE, USA), lambda phosphatase (sc-200312A) reaction mix (Santa Cruz Biotechnology, Inc., Dallas, TX, USA).

### 2.1. Generation of Monospecific pSer^312^Fet A Antibody

The peptide containing the Ser^312^ phosphorylation site of Fet A was selected based on the human Fet A protein and synthesized with and without the phosphorylated Ser^312^ (Table 1). Comparison to the rat and pig sequence showed little similarity. The peptides were synthesized as single peptides and as multiple antigen phospho-peptide (peptide) 8-(Lys)4-(Lys)2-Lys-β-Ala-OH using standard Fmoc/tBu chemistry [29] on a multiple peptide synthesizer, Syro II (MultiSynTech. Witten, Germany). The peptides were purified using preparative reversed phase-high pressure liquid chromatography and their identity was confirmed using electro spray ionization-mass spectrometry and matrix-assisted laser desorption ionization-time of flight mass spectrometry. Peptide purities were >95% as determined by analytical reversed phase-HPLC. The phospho-peptide was coupled to keyhole limpet hemocyanin using the glutardialdehyde method. The antisera were obtained after repeated immunization of 2 rabbits with a 1:1-mixture of the phospho-peptide–keyhole limpet hemocyanin conjugate and the multiple antigen phospho-peptide (Pineda Antibody-Service GmbH, Berlin, Germany). The obtained antisera from the 2 rabbits yielded similar antibody titer.

Affinity purification of antiserum pFetA was performed essentially as described previously [30]. Briefly 10 mg of phospho- or nonphospho-peptide (SLGSPpSGEVSHPRK, where pS indicates phospho-Ser) were coupled separately to 1 g of CH-activated Sepharose (GE Healthcare) according to the manufacturer’s instructions. The antiserum (5 mL) was diluted with 5 mL phosphate-buffered saline (PBS) and applied to the phospho-peptide Sepharose column (10 × 1 cm). The column was rotated overnight at 4 °C and washed extensively with PBS. The bound antibodies were eluted with 0.1 M citrate buffer (pH 3.0) and immediately neutralized with 0.5 M phosphate buffer (pH 8.0). The eluate was applied to the nonphospho-peptide Sepharose column (10 × 1 cm). After overnight rotation at 4 °C the flow-through was collected and concentrated to 0.4 mg protein/mL using a 20 kDa ultrafiltration membrane (Amicon, Merck, Darmstadt, Germany). The affinity purified pFetA serum showed no cross-reactivity with the unphosphorylated peptide as evaluated by ELISA.

### 2.2. Enzyme-Linked Immunosorbent Assay (ELISA)

ELISAs were performed as described [30,31]. Wells of 96-well plates (MaxiSorb surface (Nunc Brand products. Wiesbaden, Germany) were coated with phospho- or nonphospho-peptides (10 µg in 100 µL PBS/well) (Table 1) overnight at 4 °C in an orbital shaker. Wells were washed three times with wash buffer (WBu) (0.05% Tween 20 in PBS. pH 7.0) and then incubated with 2% bovine serum albumin (BSA) in WBu for 2 h at 37 °C. After three washes peptide-coated wells were incubated for 1.5 h at 37 °C with non-purified or purified antisera, diluted 1:20,000 or 1:100, respectively, in WBu containing 0.5% BSA. After five washes wells were incubated with horseradish peroxidase-conjugated goat anti-rabbit IgG (Dianova, Hamburg, Germany) for 1 h at 37 °C (1:2000 diluted in WBu containing 0.5% BSA). After five washes with WBu 100 µL of 1 mg/mL azino-diethylbenzthiazoline sulfonate/H_2_O_2_ in 0.1 M citrate buffer (pH 4.5) were added to each well. After 20 min at 37 °C, light absorbance was measured at 405 nm.

### 2.3. Characterization of Phospho-Ser^312^ Fet A Antibody

#### 2.3.1. Dephosphorylation of pSer^312^Fet A

For dephosphorylation of Fet A the commercial kit from Santa Cruz was used. In a total of 20 µL the reaction mix contained: 2 µL buffer, 2 µL MnCl_2_, 0.5 µL λ-phosphatase (50,000 U) supplied by the manufacturer and 1 µL 5 mM DTT, 6.5 µL H_2_O and 8 µL Fet A (80 ng) and incubated at 37 °C for the time period indicated. For Western blot analysis samples (3 µL) were diluted with 1.5 mL H_2_O and 20 µL were used for electrophoresis.

#### 2.3.2. Phosphorylation of Fet A

For in vitro phosphorylation kinase FAM20C from R&D Systems was used. The reaction mixture of 20 µL volume has contained: 1 µL kinase FAM20C, 5 µL kinase assay buffer (25 mM MOPS pH 7.2 containing 12.5 mM β-glycerol-phosphate, 25 mM MgCl_2_, 5 mM EGTA, 2 mM EDTA). 1 µL 5 mM DTT, 5 µL ATP, 8 µL human Fet A/recombinant Fet A (80 ng) and incubated at 37 °C for the time period as indicated. The gel electrophoresis sample buffer was added to stop the reaction.

#### 2.3.3. Stability of pSer^312^ Fet A

The stability of the phosphorylation of pSer^312^ Fet A was determined in freshly drawn full blood at 0 °C and at room temperature in blood collection tubes containing different additives as indicated. EDTA/protease inhibitor mix was obtained from Roche (Basel, Switzerland) sf – ss sf-ss. 

#### 2.3.4. Deglycosylation of Fet A

For the deglycosylation of Fet A the commercial protein deglycosylation Mix II (BioLabs Inc New England, Ipswich, MA, USA) was used. The de-O-glycosylation of Fet A was performed with the commercial kit from BioLabs Neuraminidase a2-3, 6, 8, Neuraminidase + O-Glycosidase and for de-N-glycosylation of fetuin A PNGase F from BioLabs Inc (New England, Ipswich, MA, USA) was used.

#### 2.3.5. Western Blot Analysis of Commercial Fet A, rh Fet A, and pFet A from Human Plasma

Plasma samples were diluted 1:500 in water. Equal amount of 5 Laemmli buffer was added and gently mixed. Samples and standards were boiled at 95 °C for 7 min and separated by sodium dodecyl sulfate polyacrylamide (7.5–19%) gradient gel electrophoresis. The proteins were transferred into Polyvinylidene difluoride (PVDF) membranes by semi-dry Western blot (transfer buffer: 48 mM Tris, 39 mM glycine, 0.0375% sodium dodecyl sulfate and 20% (*v*/*v*) methanol). After gel electrophoresis PVDF membranes were blocked with NET G buffer (150 mM NaCl, 50 mM Tris/HCl, pH 7.4, 5 mM EDTA, 0.05% Triton X-100 and 0.25% gelatin) and washed 3 times for 15 min. Then the membranes were incubated with the antibody against Fet A, pFet A (diluted 1:1000. 1:2500 in NET G) overnight at 4 °C on the rocker. After washing in NET G, the membranes were incubated with infrared (fluorescent-labeled) secondary antibody donkey anti-rabbit IR-Dye 800 CW (diluted 1:20,000 in NET G) for detection of pFet A (green color) and donkey anti-mouse IR-Dye 680 LT (diluted 1:20,000 in NET G) for detection of fetuin A (red color) for 1 h at room temperature on the rocker.

For visualization of Fet A and pFet A and the precise determination of the pFet A/Fet A ratio we used the infrared detection method with the ImageJ software (Odyssey^®^ Infrared Imaging System from LI-COR, Biosciences; Lincoln, NE, USA) which uses secondary antibodies labeled with IR-Dye near-infrared fluorescent dyes thus direct detection can be performed. One of the greatest benefits of infrared imaging is the ability to detect two protein targets in each sample lane without the need for re-probing and stripping compared to chemiluminescence mediated detection. Because of two solid state diode lasers which provide simultaneously light excitation at 685 and 785 nm (fluorescence secondary antibodies available for the 680 and 700 nm spectrum), we can quantify the total and phosphorylated form of Fet A separately in the same blot [32].

#### 2.3.6. Determination of Free Fatty Acid Composition of Fet A from Commercial Fet A and rh Fet A

Fet A (1 mg/mL) from Sigma-Aldrich or rhFet A (1 mg/mL) together with 0.2 mL internal standard were delipidated with CH_3_OH/Toluol 1:3 (*v/v*) and free fatty acids were esterified, and subsequently quantified gas chromatography as previously described [33].

### 2.4. Human Samples

Whenever blood, plasma, or serum from humans was analyzed, we took data from subjects at risk of future development of type 2 diabetes taking part of the TUebingen Lifestyle Intervention Program (TULIP). The TULIP study was designed to find parameters that predict the effect of a lifestyle intervention with diet and moderate increase in aerobic physical activity to improve prediabetes phenotypes and the cardiovascular risk profile [34,35]. The participants underwent a standard 75-g oral glucose tolerance test (OGTT). Venous plasma samples were obtained at 0, 30, 60, 90, and 120 min for determination of plasma glucose and insulin. Blood glucose was determined using a bedside glucose analyzer (glucose-oxidase method; YSI, Yellow Springs Instruments, Yellow Springs, OH, USA). The study was approved by the local research ethics committee, and written informed consent was obtained from all participants.

## 3. Results

### 3.1. In Vitro Dephosphorylation and Phosphorylation of Fet A

**Figure 1:**
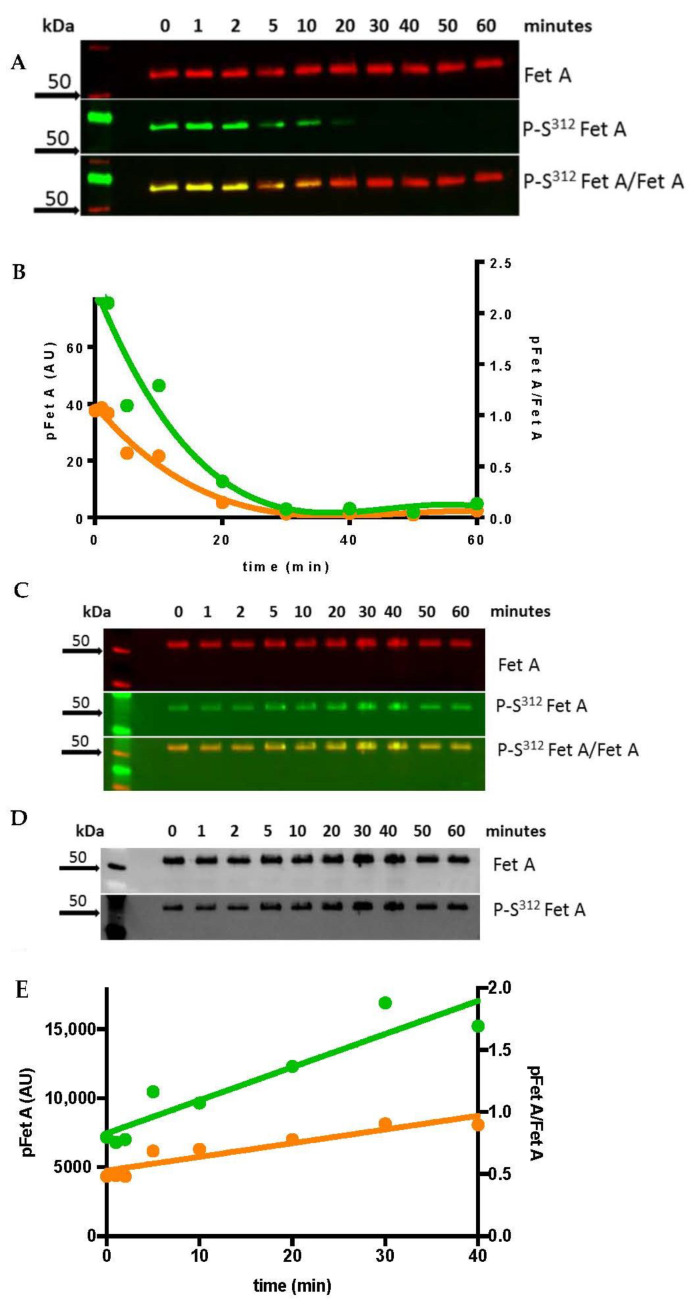
Enzymatic dephosphorylation and phosphorylation of Fet A. (**A**) Western blot analysis of time-dependent λ-phosphatase-catalyzed dephosphorylation of Fet A (red) pFet A (green) and overlay pFet A/Fet A (yellow/red). (**B**) Densitometric evaluation of Western blot data of pFet A (green) and pFet A/Fet A (orange) is shown. A representative Western blot is shown, three experiments have been performed. (**C**) Western blot analysis of time-dependent phosphorylation of commercial Fet A with FAM20C. (**D**) For comparison, the same Western blot was also developed by the conventional chemiluminescence method. (**E**) This figure shows the densitometric evaluation of the time-dependent phosphorylation of commercial Fet A. For detailed description of the procedure and the quantification see the Methods section. Bold arrows indicate molecular weight marker; AU, arbitrary units.

To prove that the generated antibody specifically recognizes pFet A but not unphosphorylated Fet A we determined the gradual decrease of phosphorylated commercial Fet A when incubated with λ-Phosphatase (Figure 1A). The signal for pFet A decreased during the first 30 min while the signal for Fet A was essentially unchanged during the whole incubation time. Quantitative analysis confirmed the decrease of the pFet A/Fet A ratio (Figure 1B). The overlay clearly indicates a relative decrease of pFet A by changing the color from yellow via orange to red. Since the detection with the different antibodies is performed on the same run the overlay represents the pFet A/Fet A ratio. The Ser^312^ antibody did not detect any phosphorylation of untreated rhFet A. Besides Fet A, the related form Fet B is expressed by the human liver [36]. Therefore, we tested if Fet B may be also detected by our antibody. Not unexpectedly, no phosphorylation of Fet B was detected with our Ser^312^ antibody, since the consensus phosphorylation sites of Fet A are not present in Fet B.

To understand the possible function and regulation of hepatic Fet A and pFet A synthesis and secretion we searched for the kinase responsible for the phosphorylation. Since in Hep G2 cells, FAM20C kinase-mediated Fet A phosphorylation has been found, we performed in vitro phosphorylation assays [37]. Phosphorylation of commercial Fet A with kinase FAM20C showed a time-dependent increase of pFet A, as also shown by the overlay representing the pFet A/Fet A ratio (Figure 1C). The same Western blot was also developed by the conventional chemiluminescence method for comparison of the new and the conventional method (Figure 1D). Quantitative analysis indicated a steady increase of the pFet A/Fet A ratio up to 85% above basal values within 60 min (Figure 1E), suggesting that this Fet A preparation is not completely phosphorylated at Ser^312^ under basal conditions. No Fet A phosphorylation could be detected when Fet A was incubated with glycogen synthase kinase. Altogether, these results support the specificity of the generated antibody for the pSer^312^ Fet A. Furthermore, our assay, due to the overlay technic, is suitable to detect small changes in pFet A/Fet A ratio.

### 3.2. Stability of pSer^312^ Fet A in Human Blood Samples

**Figure 2:**
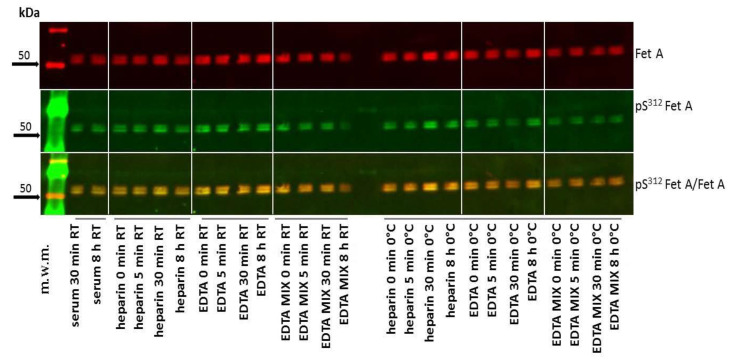
Stability of pSer^312^ Fet A in human blood samples collected in different routine blood tubes at Western blot analysis of blood samples from a healthy volunteer and the stability of pFet A was evaluated in sample material used in routine clinical chemistry. Blood samples were collected in tubes containing different additives i.e., heparin, EDTA, and EDTA mixture as described in the method section. Bold arrows indicate molecular weight marker (m.w.m).

Since human plasma and serum contains numerous phosphatases, we studied the conditions under which pSer^312^ Fet A is stable in patients’ samples. Therefore, we collected blood in tubes containing different additives that are used in routine blood sampling. Our data shows that pSer^312^ Fet A is unchanged in serum, heparin-, EDTA-blood and in blood with EDTA/protease inhibitor mix at 0 °C and room temperature for up to 8 h (Figure 2). These results indicate that the pSer^312^ phosphorylation of Fet A is not sensitive to temperature and is not dependent on time left before analysis and sample material used in routine blood sampling.

### 3.3. Precision of the Developed Assay

**Figure 3:**
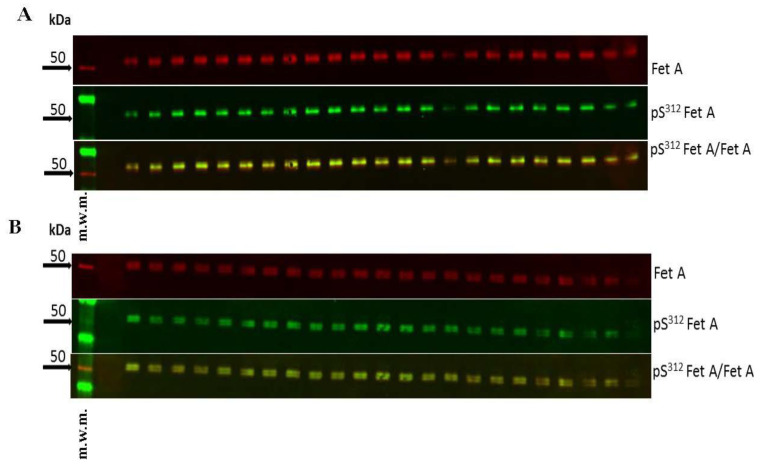
Assay variations of two subjects with different BMI. Representative Western blots of Fet A, pFet A and overlay from two plasma samples from subjects with (**A**) BMI of 20 kg/m^2^ and (**B**) BMI of 35 kg/m^2^. To evaluate precision of the analysis 23 samples of each individual were run in one assay. For evaluation of the assay variation the last 3 lanes were not included for the calculation of variation because of unproper run of these samples. Western blot analysis and densitometric evaluations were performed as described in the Method section. m.w.m = molecular weight marker.

For determination of the imprecision, 23 plasma samples of two individuals with low (20 kg/m^2^) and high (35 kg/m^2^) BMI were analyzed by Western blotting in one run (Figure 3A,B). Precision analysis was performed as described previously [38]. The coefficients of variation were 14%, 19% and 11% (BMI 20 kg/m^2^); and 13%, 24% and 17% (35 kg/m^2^) for Fet A, pFet A and the ratio pFet A/Fet A, respectively (Table 2). Figure 3 also indicates that different subjects in this case with low and high BMI show different Fet A microheterogeneities. Only one band Fet A is present in the Western blot of the first individual (Figure 3A) while two bands are obvious in the Western blot of the second individual (Figure 3 B). Figure 3B clearly indicates that both Fet A forms are phosphorylated at Ser^312^.

### 3.4. Effect of Deglycosylation of Fet A on pSer^312^

**Figure 4:**
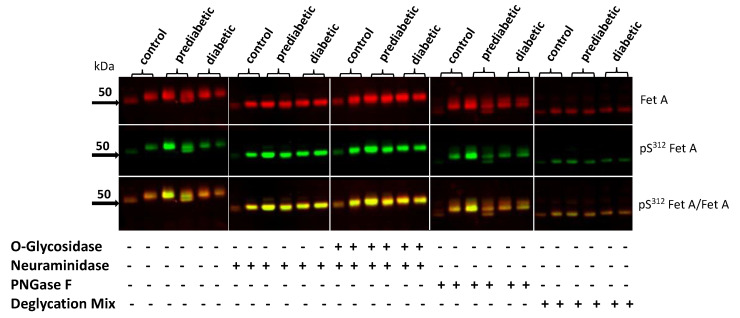
Deglycosylation of Fet A from control, prediabetic, and diabetic patients and effect on Ser^312^ Fet A phosphorylation. Serum sample from control, prediabetic and diabetic patients (two of each) were analyzed untreated or treated with the deglycosylation enzymes as indicated. Samples were analyzed for Fet A, pFet A, and overlay by Western blotting as described in the Methods section; bold arrows indicate molecular weight markers.

Previous reports indicate that Fet A is glycosylated, which may be responsible for the microheterogeneity [39] suggesting a possible influence of Fet A glycosylation on the phosphorylation of Fet A. To evaluate this hypothesis, we analyzed the effect of different deglycosylation enzymes on pSer^312^ of Fet A. We used serum sample of two controls, two prediabetic, and two diabetic individuals for our analysis. The untreated samples showed variable glycosylation and phosphorylation pattern in the Western blot (Figure 4). Upon treatment with neuraminidase the pattern appeared less variable and the pFet A/Fet A ratio was similar except one control sample (left) showing lower signals due to an edge zone effect. Only treatment with the deglycosylation mix containing O- and N-Glycosidase and neuraminidase yielded one small band at approximately 47 kD. The data indicates that deglycosylation does not affect pSer^312^ phosphorylation of Fet A. Furthermore, the data suggests that non-diabetic, prediabetic, and diabetic subjects seem not to display specific Fet A patterns in Western blotting.

### 3.5. Associations of the Phosphorylation and Glycosylation Pattern of Fet A

**Figure 5:**
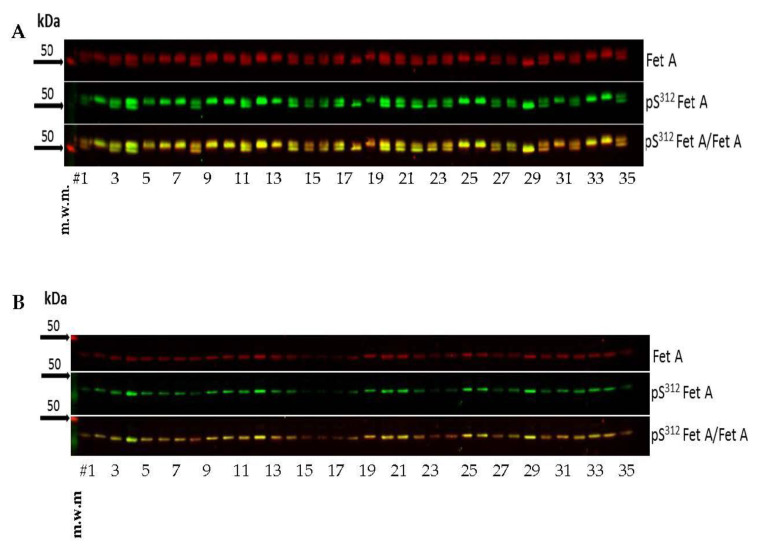
Analysis of plasma pSer^312^ pFet A and Fet A samples obtained from non-diabetic individuals (#1–35) Untreated (**A**) and completely deglycosylated (**B**) samples obtained from non-diabetic individuals (*n* = 35) were analyzed by Western blotting as described in the Method section; m.w.m = molecular weight marker.

To find out if glycosylation and phosphorylation of Fet A are associated we analyzed 35 non-diabetic subjects (14 women and 21 men, age 51.0 [42.0–55.0] years; weight 90.5 [86.5–101.5] kg; BMI 30.82 [28.89–34.40] kg/m^2^; fasting glucose 5.22 [4.83–5.78] mM; fasting insulin 48 [35.7–80] pM; HOMA 1.38 [1.22–2.48]; insulin sensitivity (OGTT) 10.84 [8.08–13.05] 10^19^ × l^2^ × mol^−2^; data are shown as median [interquartile range]). Untreated samples show very different glycosylation and phosphorylation patterns (Figure 5A) with double or single bands. When the same samples were treated with the deglycosylation MIX II (Figure 5B) only one single band remained indicating the deglycosylated Fet A species. It follows that the upper band in Figure 5A indicates higher glycosylation while the lower band indicates less glycosylation, and that the investigated subjects show double or upper and lower single bands reflecting the large variation of glycosylation. Evaluating the intensity of the single bands and the overlay, high phosphorylation of Fet A can be observed in the bands of subject # 4 (double), # 12 (upper), #2 5 + # 26 (upper) and # 29 (lower). After deglycosylation the intensity of the overlay remained high. Inversely, low phosphorylation of Fet A can be observed in the bands of subject # 8 (double), # 12 (upper), # 27, # 28 and # 30 (all double). Accordingly, the intensity of the overlay remained low after deglycosylation. Together these data suggest that the phosphorylation of Fet A is independent of the glycosylation status of Fet A.

Since a previous report [6] indicated that pSer^312^ pFet is elevated in obese individuals when compared to normal individuals, we quantified the upper and the lower band and the sum of both pSer^312^ pFet A signals shown in Figure 5A and the respective deglycosylated Fet A forms shown in Figure 5B. As demonstrated in Table 3 we found no significant correlation between either these anthropometric and metabolic parameters and pSer^312^ pFet A. We consider the statistically significant gender difference in pFet A (2) to be rather a chance finding.

### 3.6. Short-Term Metabolic Changes (OGTT) Do Not Affect Fet A or pFetA Plasma Levels

**Figure 6:**
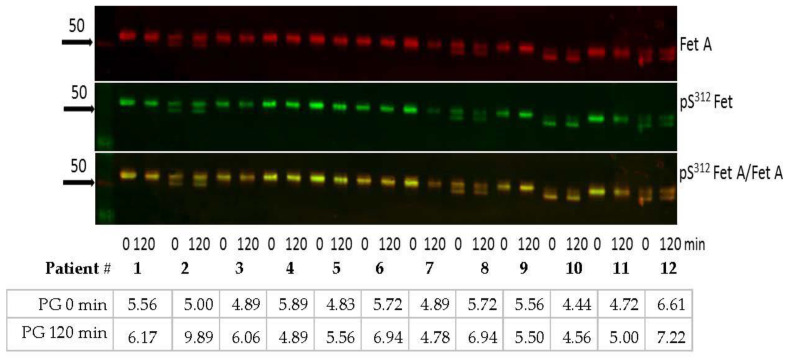
Effect of short-term metabolic changes on plasma pSer^312^ pFet A and Fet A induced by an OGTT. Serum samples were obtained from 12 controls during an OGTT and sample taken at 0 min (basal) and 120 min were analyzed by Western blotting as described in the Method section. PG: Plasma glucose in mmol/L. Bold arrows indicate molecular weight markers.

To evaluate if short-term changes in metabolism affects pFet A/Fet A ratio; we studied serum samples of 12 non-diabetic subjects during an OGTT. As shown in Figure 6, no significant change of the pFet A/Fet A ratio was found between basal and 120 min OGTT. Furthermore, the analysis indicates that both the glycosylation and the phosphorylation pattern remain unchanged after 120 min of a 75 g glucose load. These results indicate no short-term effects of metabolic changes i.e., elevated glucose and/or insulin levels on the Fet A phosphorylation. However, Figure 6 indicates an individual Fet A/pFet A pattern. For example, subjects 2, 8, 10, and 12 show double bands, which remain unchanged during the OGTT. Although these double bands are similarly stained for Fet A, pFet A and pFet A/Fet A ratio in patient 2, 8, and 12, staining of patient 10 shows that the lower band is more abundant and more phosphorylated again indicating microheterogeneity of Fet A and pFet A.

### 3.7. Determination of Fatty Acid Composition of Fet A

**Figure 7:**
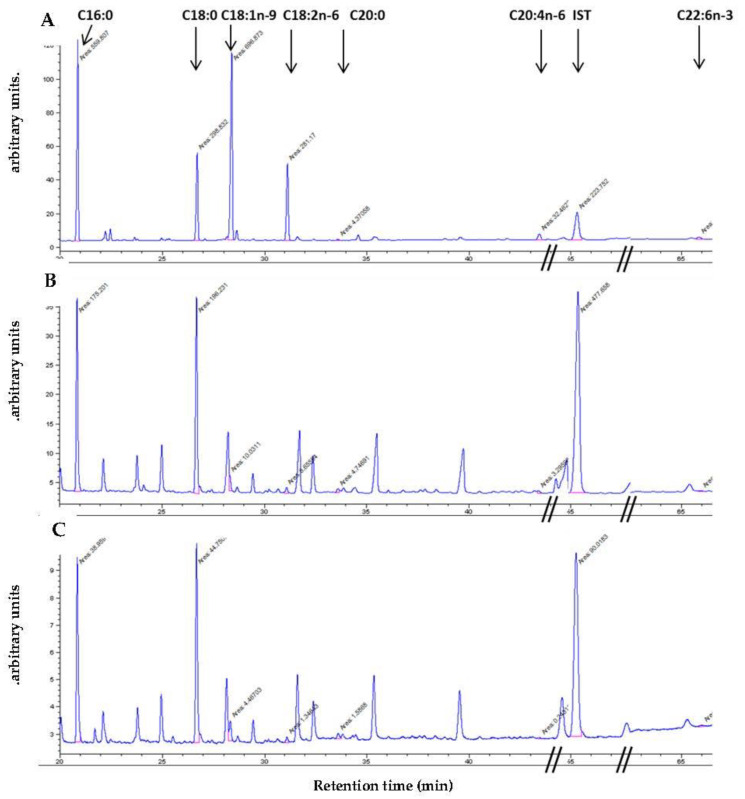
Free fatty acid analysis of human serum and Fet A preparation. Free fatty acid pattern of a representative human serum, Fet A from Sigma-Aldrich, and rhFet A were analyzed as described in the Method section. (**A**) A gas chromatogram of the fatty acid pattern of a serum from a representative healthy individual is shown. (**B**,**C**) show chromatograms of the fatty acid pattern of commercial Fet A and of rhFet A, respectively. 16:0, 18:0 and 20:0 indicate the saturated fatty acids palmitate, stearate and arachinate; 18:1 indicates the monounsaturated fatty acid oleate and the poly-unsaturated fatty acids as indicated by 18:2 = linoleate, 20:4 = arachidonate and 22:6 = docosahexaenoic acid, respectively; IST = internal standard. Since the areas of the respective peaks are not readable in the original chromatograms they are listed below (arbitrary units): 16:0 559.8, 175.2, 38.95; 18:0: 295.8, 196.2, 44.75; 18:1: 696.8, 10.0, 4.47; 18:2: 281.2 6.65, 1.34; 20:0: 4.37, 4.75, 1.58; 20:4: 32.5, 3.29, 0.25; IS: 223.7, 447.7, 90.0 and 22:6: 17.9, 1.3, 0.71 for chromatogram A, B, and C, respectively.

Free fatty acid pattern of Fet A from Sigma-Aldrich and rhFet A were analyzed as previously described [40]. A representative chromatogram of the fatty acid pattern of serum free fatty acid fraction is shown in Figure 7A for comparison with the fatty acid pattern found in commercial Fet A and rhFet A, Figure 7B,C, respectively. The results of the quantitative analysis are shown in Table 4. Although the amount of saturated free fatty acids (16:0, 18:0 and 20:0) sum up to 45.64% in human serum, the corresponding values for commercially obtained Fet A and rhFet A are 94.64% and 92.68%, respectively. The respective values for the monounsaturated fatty acid oleate (18:1n-9) are 36.86, 2.52 and 4.88%. The poly-unsaturated fatty acids (18:2n-6, C20:4n-6, 22:6n-3) sum up to 17.55%, 2.85% and 2.44% for human serum, commercially obtained Fet A and rhFet A, respectively. The respective ratios for saturated fatty acids/monounsaturated are 1.23, 37.5 and 19.3 and the ratios for saturated fatty acids/poly-unsaturated fatty acids are 0.84, 17.6 and 12.7, respectively. Together these results indicate a more than 10-fold preferential binding of saturated fatty acids vs. unsaturated fatty acids to Fet A and rhFet A compared to the abundance of saturated free fatty acids occurring in human serum.

## 4. Discussion

Based on previous results showing that only the phosphorylated but not unphosphorylated Fet A inhibits insulin signal transduction, we hypothesized that the serum or plasma pFet A may be the biological form of Fet A exerting its metabolic function. We therefore set out to find a method to detect and to characterize pFet A. To achieve this goal, we generated antibodies directed against Fet A phosphorylated at Ser^312^, which is the most abundantly phosphorylated site. The antibody recognized a time-dependent enzymatic dephosphorylation of pFet A as well as rephosphorylation of Fet A with FAM20C supporting the specificity of the generated antibody. Furthermore, the finding that commercial Fet A isolated from human serum could be further phosphorylated confirms a previous report that Ser^312^ is not fully phosphorylated [5]. Since Fet A may be glycosylated and may occur in different forms in human plasma [6], we used Western blotting for identification and quantification of pFet A and Fet A to evaluate if the modifications may possibly influence each other. Furthermore, using the Infrared Imagining System (see Methods) for the visualization, we could detect both pFet A and Fet A in one run and, by overlay, visualize the pFet A/Fet A ratio of the individual Fet A species differing in glycosylation pattern. Applying this method, we demonstrated that Fet A occurs in different forms in the individual subject studied and that these forms may be differently phosphorylated. Therefore, we deglycosylated the samples to obtain a homogenous Fet A. The samples were analyzed for their amount of Fet A phosphorylation. Taken together, this assay protocol can be used to assess the effect of protein modifications/glycosylation on protein phosphorylation or vice versa.

In our search for the physiologic kinase responsible for Fet A phosphorylation we screened for kinases with the Ser^312^ consensus site in Fet A. We found, besides other candidates, glycogen synthase kinase, which however did not phosphorylate Fet A at Ser^312^ in an in vitro assay. Then we tested FAM20C kinase and found that this kinase phosphorylates Fet A on Ser^312^ time-dependently. Previous reports have indicated that FAM20C is a single kinase generating most of the secreted phospho-proteome [37]. FAMC20-mediated phosphorylation of the secreted proteins takes place in the lumen of the Endoplasmatic Reticulum and Golgi apparatus. Besides liver, FAM20C is also highly expressed in lactating mammary gland and mineralized tissues. Accordingly, studies with cultured breast cancer cells indicate that depletion of FAMC20 resulted in dramatic effects on cell adhesion, migration, and invasion. Furthermore, deletion of FAM20C in whole animals induces a syndrome resembling the human Raine syndrome thus pointing to a function in the extracellular matrix/collagen/Calcium metabolism [37]. However, no effects on carbohydrate or lipid metabolism have been reported in these studies. FAM20C-dependent phospho-sites have been found in numerous secretory pathway proteins including Fet A from HepG2 cells but, to the best of our knowledge, phosphorylation of human Fet A by FAM20C has not been shown [37]. Thus, the biological relevance of FAM20C dependent Fet A phosphorylation in humans remains to be elucidated.

Before applying our assay to human blood species, we evaluated the performance of our novel assay format. Since preanalytical issues may influence the results, we studied the stability of pFet A in different routinely used sample materials. We found that pFet A levels were unchanged in serum or EDTA plasma over time (8 h) at 0 °C and at room temperature (Figure 2). This finding indicates that intrinsic phosphatases such as alkaline or acidic phosphatases are not active under these conditions and that pFet A is stable under routine clinical blood taking conditions. Furthermore, we found no short-term metabolic effects on pFet A since samples from an OGTT, (0 and 120 min) were not different (Figure 6). These results together with the findings that the developed assay is precise suggest that the assay format is suitable for determination of pFet A in human subjects.

Although a previous study [6] has reported that the extent of Ser^312^ phosphorylation of Fet A is associated with BMI, serum glucose, serum insulin and HOMA-IR we did not observe any significant correlation between these anthropometric and metabolic parameters and pFet A forms (Table 3). The apparent differences may be explained by the different patients’ characteristics in theirs and our study. In particular, in their study also extremely obese individuals were included compared to our cohort. This difference is reflected by a considerable proportion of patients with higher BMI, higher insulin levels and particularly elevated insulin resistance as assessed by the HOMA-IR index. It may thus well be that the significant correlation with pFet A is, to a large extent, driven by these grossly obese individuals included in their cohort.

Our assay format also indicates that different forms of pFet A, particularly variably glycosylated forms, are present in human plasma. Considering that the biosynthesis of the Fet A protein takes place in the rough Endoplasmatic Reticulum before the phosphorylation is achieved in the Golgi apparatus, it may well be that the glycosylation pattern governs the phosphorylation of Fet A. However, our results indicate that Fet A phosphorylation is independent from the glycosylation status. Therefore, deglycosylation of human sample before measuring Fet A and pFet A, respectively, may be indicated to determine a well-defined molecular form of Fet A and pFet A. Furthermore, our results may stimulate the analytical improvement of other multi-modified laboratory parameters by e.g., deglycosylation to obtain well-defined analytes on the molecular level.

Since previous reports strongly suggest that Fet A and palmitate act synergistically in promoting inflammatory response [28], the fatty acid pattern bound to Fet A may be responsible for its biological effects. Therefore, we analyzed the fatty acid pattern bound to Fet A. We found that commercially available Fet A-bound fatty acid pattern is strongly shifted to the presence of saturated fatty acid pattern (>90%) while oleate and linoleate are reduced by more than 10-fold. Similarly, rhFet A showed an according preferential binding of saturated fatty acids at the expense of mono- and poly-unsaturated fatty acids. Together with our finding that rhFet A is unphosphorylated, these data indicate that Ser^312^ phosphorylation has no or little effect on the fatty acid pattern bound to Fet A.

A previous study by Cayatte et al. [41] comparing bovine albumin and bovine Fet A-bound fatty acid pattern observed a far less pronounced difference in fatty acid binding. When compared to bovine albumin, bovine Fet A binds more saturated FFA (55.7 vs. 35.3%), binds less oleate (27.1 vs. 31.9%) and binds much less linoleate (3.9 vs. 16.2%). Therefore, the effect of this Fet A “modification” needs to be further investigated in future studies.

Our current knowledge indicates that Fet A exerts diverse actions apparently diametrically opposed: for example, elevated Fet A levels lead to impaired metabolism, while lack of Fet A leads to massive ectopic, particular vascular calcification [42]. It is currently unclear how Fet A can act in such diverse ways. It may well be that Fet A modifications such as glycosylation, phosphorylation, and fatty acid binding may govern the different functions of Fet A, e.g., by determining its organ-specific action. Although current data suggest an effect of Fet A phosphorylation on metabolism, any effect of this Fet A modification on tissue calcification is unknown. To the best of our knowledge, the biological effects of Fet A glycosylation have not been studied. However, previous reports show that palmitate is a potent coactivator of Fet A in activating TLR4 leading to increased pro-inflammatory cytokine production [28,43,44]. Therefore, our finding that human Fet A preferentially binds saturated fatty acids indicates that this modification assigns Fet A to pro-inflammatory properties.

Our results, together with previous data, indicate that the biological effects of Fet A are modulated by modification of Fet A. The biological effects of Fet A may contribute to the vascular complications of diabetes by at least two mechanisms: (i) the established property of Fet A, particularly phosphorylated Fet A, to inhibit insulin signaling, thus promoting insulin resistance in humans [6,25]. Previous reports have shown that elevated insulin resistance is associated with macrovascular, and particularly cardiovascular disease [16,18]. This association is supported by the finding that elevated Fet A serum levels indicate increased liver fat [14], which in turn has also been shown to be associated with increased cardiovascular disease [45]; (ii) the ability of Fet A together with saturated fatty acids e.g., palmitate to stimulate a pro-inflammatory cascade via activating toll-like receptor 4, thus promoting endothelial dysfunction and vascular disease [15,28].

In conclusion, we have developed a Western blot-based immunoassay for the detection and quantification of both Fet A and pFet A phosphorylated at Ser^312^ in one assay. The assay is specific and can be used for the detailed investigation of pFet A in human serum or plasma. We found that Fet A as well as pFet A are heterogeneous in human samples due to different glycosylation, and that glycosylation does not affect phosphorylation of Fet A. Other biochemical modifications, including fatty acids bound to Fet A may be more important for determining the biological effects of Fet A, and thus, its contribution to the pathophysiology of the chronic complications of diabetes.

## Figures and Tables

**Table 1:** Partial Fetuin A sequence alignment of different species (human; pig and rat). The human phospho-peptide was used to generate antibodies against phospho-Ser^312^ Fet A (pFet A), bold **S** indicates Ser^312^ in human Fet A.

Species	Fet A Sequence Alignment
human	-slgsp**S**gevshprk-
pig	-svesasgeafhvgk-
rat	-svesasgevlhspk-

**Table 2:** Evaluation of assay variation of the densitometric analysis of Western blots from Figure 3A,B.

	BMI 20 kg/m^2^			BMI 35 kg/m^2^		
	Fet A (AU)	pFet A (AU)	pFet A/FetA	Fet A (AU)	pFet A (AU)	pFet A/FetA
Mean	14,222	10,647	0.75	16,702	11,681	0.697
SD	2031	1989	0.082	2189	2746	0.119
SEM	454	445	0.018	489	614	0.026
CV	14%	19%	11%	13%	24%	17%
N	20	20	20	20	20	20

SD, standard deviation; SEM, standard error; CV, coefficient of variation; AU, arbitrary units.

**Table 3:** Univariate associations of densitometric results obtained from Western blots of 35 individuals (Figure 5) for pFet A, band (1) and band (2), and deglycosylated Fet A.

	pFet A (1)		pFet A (2)		pFet A Sum		pFet A Deglyco	
	r	*p*	r	*p*	r	*p*	r	*p*
Gender	0.21	0.22	0.42	0.01	0.18	0.23	0.32	0.06
Age	−0.14	0.43	+0.10	0.58	+0.02	0.92	+0.08	0.63
BMI	+0.17	0.33	−0.03	0.86	+0.14	0.42	+0.10	0.56
Fasting glucose	+0.08	0.22	+0.06	0.73	+0.01	0.95	+0.14	0.42
2 h glucose _OGTT_	−0.05	0.77	+0.15	0.39	+0.11	0.54	+0.29	0.09
Fasting insulin	+0.03	0.88	+0.08	0.66	−0.08	0.64	+0.30	0.08
2 h insulin _OGTT_	+0.12	0.49	+0.12	0.49	−0.00	0.97	+0.30	0.08
HOMA-IR	+0.04	0.82	+0.08	0.63	−0.08	0.67	+0.31	0.07
Insulin sensitivity _OGTT_	−0.11	0.51	−0.10	0.56	+0.01	0.94	−0.27	0.11

BMI, body mass index; OGTT, oral glucose tolerance test; HOMA-IR, homeostasis model assessment of insulin resistance. pFet A, phosphorylated fetuin A; deglyco, deglycosylated. (1) and (2) refer to upper and lower bands of pFet A and sum refers to the sum of these bands.

**Table 4:** Free fatty acid pattern of human serum from a healthy individual and Fet A preparations. Percentages of the main free fatty acids of human serum, Fet A from Sigma-Aldrich, and rhFet A are shown.

Fatty Acid Species	FFA Plasma Sample	Commercial Fet A	rh Fet A
C16:0	29.61	44.09	42.32
C18:0	15.80	49.37	48.62
C18:1N9 (MUFA)	36.86	2.52	4.88
C18:2N6	14.87	1.69	1.41
C20:0	0.23	1.18	1.74
C20:4N6	1.72	0.83	0.26
C22:6N3	0.91	0.33	0.77
SFA (sum)	45.64	94.64	92.68
PUFA (sum)	17.55	2.85	2.44
MUFA/SFA	1.23	37.5	19.3
uSFA/SFA	0.84	17.6	12.7

Free fatty acids = FFA, saturated free fatty acids = SFA, monounsaturated free fatty acids = MUFA, poly-unsaturated free fatty acids = PUFA, sum of unsaturated free fatty acids = uSFA.

## Data Availability

Original data are available upon request.
